# First growth curves based on the World Health Organization reference in a Nationally-Representative Sample of Pediatric Population in the Middle East and North Africa (MENA): the CASPIAN-III study

**DOI:** 10.1186/1471-2431-12-149

**Published:** 2012-09-17

**Authors:** Marjan Mansourian, Hamid Reza Marateb, Roya Kelishadi, Mohammad Esmaeil Motlagh, Tahereh Aminaee, Mahnaz Taslimi, Reza Majdzadeh, Ramin Heshmat, Gelayol Ardalan, Parinaz Poursafa

**Affiliations:** 1 Department of Biostatistics and Epidemiology, Health School, Isfahan University of Medical Science, Isfahan, Iran; 2 Biomedical Engineering Department, Engineering Faculty, the University of Isfahan, Isfahan, Iran; 3 Pediatrics Department, Child Growth and Development Research Center, Isfahan University of Medical Sciences, Isfahan, Iran; 4 Department of Pediatrics, Ahvaz Jundishapur University of Medical Sciences, Ahvaz, Iran; 5 Bureau of Family Health, Ministry of Health and Medical Education, Tehran, Iran; 6 Bureau of Health and Fitness, Ministry of Education, Tehran, Iran; 7 Epidemiology Department, School of Public Health, and Knowledge Utilization Research Center ,Tehran University of Medical Sciences, Tehran, Iran; 8 Endocrinology& Metabolism Research Institute, Tehran University of Medical Sciences, Tehran, Iran; 9 Environment Research Center, Isfahan University of Medical Sciences, Isfahan, Iran

**Keywords:** Growth, Iran, Reference curve, Weight disorder

## Abstract

**Background:**

The World Health Organization (WHO) is in the process of establishing a new global database on the growth of school children and adolescents. Limited national data exist from Asian children, notably those living in the Middle East and North Africa (MENA). This study aimed to generate the growth chart of a nationally representative sample of Iranian children aged 10–19 years, and to explore how well these anthropometric data match with international growth references.

**Methods:**

In this nationwide study, the anthropometric data were recorded from Iranian students, aged 10–19 years, who were selected by multistage random cluster sampling from urban and rural areas. Prior to the analysis, outliers were excluded from the features height-for-age and body mass index (BMI)-for-age using the NCHS/WHO cut-offs. The Box-Cox power exponential (BCPE) method was used to calculate height-for-age and BMI-for-age Z-scores for our study participants. Then, children with overweight, obesity, thinness, and severe thinness were identified using the BMI-for-age z-scores. Moreover, stunted children were detected using the height-for-age z-scores. The growth curve of the Iranian children was then generated from the z-scores, smoothed by cubic S-plines.

**Results:**

The study population comprised 5430 school students consisting of 2312 (44%) participants aged 10–14 years , and 3118 (58%) with 15–19 years of age. Eight percent of the participants had low BMI (thinness: 6% and severe thinness: 2%), 20% had high BMI (overweight: 14% and obesity: 6%), and 7% were stunted. The prevalence rates of low and high BMI were greater in boys than in girls (P < 0.001). The mean BMI-for-age, and the average height-for-age of Iranian children aged 10–19 years were lower than the WHO 2007 and United states Centers for Disease Control and Prevention 2000 (USCDC2000) references.

**Conclusions:**

The current growth curves generated from a national dataset may be included for establishing WHO global database on children’s growth. Similar to most low-and middle income populations, Iranian children aged 10–19 years are facing a double burden of weight disorders, notably under- and over- nutrition, which should be considered in public health policy-making.

## Background

Growth curves are used for screening, surveillance and monitoring of children and adolescents’ health; and are cost-effective in detecting nutritional disorders [1]. These references serve as a tool for static and dynamic diagnosis of growth disorders, tracking the growth in surveillance systems and analysis and reporting growth data and trends in various populations [2].

Adolescence, a stage of transition between childhood and adulthood, is a critical period characterized by a growth spurt with physiological changes until reaching approximate adult status [3]. However, in comparison with the period of early childhood, there is much more individual variation in the growth velocity, which is important in terms of crucial normality. Although growth pattern is mainly determined by genetic factors, it also reflects the nutritional status. Therefore, evaluation of nutritional disorders such as stunting (a measure of chronic under-nutrition), thinness (under-nutrition) and overweight (over-nutrition) are of essential importance.

It is well-documented that overweight has many lifelong health hazards, notably on chronic non-communicable diseases (CNCDs) [4,5]. Stunting and thinness on the other hand as under-nutrition indicators are attributable to many factors such as low birth weight [6], lack of care [7], insufficient nutrition and other environmental parameters. They capture the multiple dimensions of children's health, environment, nutritional status and development of their living place [8]. Poor growth in early life is associated with long-term impact on morbidity and mortality in later life [9]. This is of special concern among adolescent girls, as future mothers, and the fetal programming of CNCDs for the next generation [8,10,11].

Two growth references of the World Health Organization (WHO 2006/2007) [12,13] and the US Centers for Disease (USCDC2000) [14] have been widely used to calculate the anthropometric indicators. While WHO references were based on the Multi-center Growth Reference Study (MGRS) from six cities in different countries, USCDC2000 is based on the US national surveys of children aged 0–20 years that might not be appropriate for use in other populations, in particular, developing countries [15].

In April 2006 the WHO released the standards for assessing the growth and development of children from birth to 5 years of age, known as “WHO reference 2006” [12,16]. Also, WHO provided the new curves for school-aged children and adolescents in 2007 to fill the gap in growth curves and provide an appropriate reference for the 5 to 19 years age group, entitled as “WHO reference 2007” [13].

Both the WHO 2007 and USCDC2000 growth references were based mainly on cross-sectional samples with sparse longitudinal data [13,14]. They have been developed based on different principles and data sets and have provided different cut-points for the same anthropometric measures; they could, thus, provide different results. For instance, WHO 2007 recommendation is mainly based on Z-scores, while USCDC2000 prefers percentile of anthropometric measures.

The WHO Department of Nutrition is in the process of establishing a new global database on the growth of school children and adolescents by using national data from various countries. Limited national data exist from Asian children, notably from those living in the Middle East and North Africa (MENA). Although some growth references have been established in Iran in recent years, sampling was restricted to the small populations of major cities [17,18] whereas, in some cases, sampling was not random or limited as to report the percentage of every abnormal categories without constructing the references comparing to growth references and standards [19,20]. Therefore, this study aimed to generate the growth chart of a nationally- representative sample of Iranian children, and to assess how well these growth references match with, or diverge from, international growth charts.

## Methods

### Sample

The national survey of school student high risk behavior (2009–2010) was conducted as the third survey of the school-based surveillance system entitled **C**hildhood &**A**dolescence **S**urveillance and **P**revention of **I**ranian **A**dult **N**on communicable disease (CASPIAN-III) ^a^*Study [21]. We have previously reported the details of data collection and sampling of the current survey [22]; thus herein we present it in brief. This school-based nationwide health survey was conducted in Iran with corporation of the Ministry of Health and Medical education, Ministry of Education and Training, Child Growth and Development Research Center, Isfahan University of Medical Sciences, and Endocrinology and Metabolism Research Institute of Tehran University of Medical Sciences. The study was conducted according to the declaration of Helsinki [23] and approved by the institutional review boards of the Ministry of Health and Medical Education and the Ministry of Education and Training, as well as the collaborating universities at provincial level.

The project was performed among 5570 students, aged 10–19 years, selected by multistage random cluster sampling from urban and rural areas of 27 provinces of Iran. Eligible schools for our study were stratified and randomly selected according to the information bank of the Ministry of Education and Training. In the selected schools, students were also chosen randomly. Those students with the history of any chronic disease and or prolonged use of medications, as well as those on special diets or with any disability were not recruited. Eligible students were enrolled to the survey after complete explanation of the objectives and protocols. Written informed consent and oral assent were obtained from parents and students respectively.

### Measurements

A team of trained health care professionals recorded information and measured weight and height by using calibrated instruments under standard protocol recommended by the WHO [24]. Weight was measured to the nearest 100 grams in barefoot and in lightly dressed condition. Standing height in centimeters was marked on a stadiometer, barefoot, standing with heels together with 60 degree angle between feet and head was placed in the Frankfurt plane so that the line of vision was perpendicular to the body. Height was recorded to the nearest 0.1 cm. Body mass index (BMI) was then calculated as weight (kg) divided by height squared (m^2^).

According to the WHO, weight-for-age is not a suitable indicator for children older than 10 years [13]. Accordingly, two features height-for-age and BMI-for-age were assessed for our study participants. BMI-for-age is the recommended indicator for assessing thinness, severe thinness, overweight and obesity as critical problems in individuals aged 10–19 years, while height-for-age is used as the indicator for stunting [13].

### Outlier detection

Since the results of the analysis can be quite sensitive to outliers, they were deleted based on the 1977 National Center for Health Statistics and Word Health Organization (NCHS/WHO) guideline according to fixed exclusion range, as raw height-for-age Z-scores lower than −5.0 or higher than +3.0 and raw BMI-for-age z-scores lower than −4.0 or higher than +5.0 [25]. Based on the WHO criteria, 1.5% of the data set was outlier; thus excluded from the analysis.

### Statistical analysis

The Box-Cox power exponential (BCPE) distribution was fitted to the BMI-for-age and height-for-age data [13]. This was necessary to compute the BMI-for-age and height-for-age Z-scores and their comparison with those of WHO reference 2007. In fact BCPE provides a model for a dependent variable exhibiting both skewness and kurtosis [26]. The distribution has four parameters and is denoted BCPE (μ, σ, ν, τ). The parameters μ, σ, ν, and τ, may be interpreted as relating to location (median), scale (approximate coefficient of variation), skewness (transformation to symmetry) and kurtosis (power Exponential parameter), respectively. BCPE can be ultimately simplified to LMS method [27], when it is not necessary to model the kurtosis. Since the LMS method is also used to construct the USCDC2000 growth curves, the age- and sex- specific LMS values are provided on the WHO web site (http://www.who.int/growthref/). In this case, the Z-score (z) for a given height, and BMI measurement (X) can be calculated as: z = ((X/M)^L-1)/L/S if L ≠ 0 or z = log(X/M)/S if L = 0 [27].

In this project, the WHO z-score cut-offs used were as follow: 1) using BMI-for-age Z-scores; overweight: > + 1SD i.e. Z-score > 1 (equivalent to BMI 25 kg/m^2^ at 18 years), obesity: > + 2SD (equivalent to BMI 30 kg/m^2^ at 18 years), thinness:<−2SD and severe thinness: <−3SD ; 2) Stunting was defined as height-for-age < −2SD.

To produce the smooth growth curve, the smoothing cubic Spline was used [27]. In this method, the optimal number of knots, and the knots location were selected according to [28]. Furthermore, to avoid over-fitting, “wiggliness” penalty function was used which will be large if the smoothed function is very wiggly and small if it is nearly flat [29].

The normality of distributions of estimated height z-scores were assessed with the Kolmogorov-Smirnov test. Then, the mean differences of the z-scores were analyzed with the Student’s t-test for the whole age range. Differences in the prevalence of different categories between genders were tested with the Chi-square test. The analysis was performed using WHO AnthroPlus [30] and Matrix Laboratory (MATLAB) (ver. 7) softwares.

## Results

The data of 5430 students (50% boys) were complete for the current analysis, and are reported. A total of 2312 (44%) participants were in the 10-14-year- age group and 3118 (58%) in the15-19 year- category. The mean weight and height of the study population were 47.73 kg (2SD = 14.61) and 154.90 cm (2SD = 13.35), respectively. The mean BMI was 19.49 kg/m^2^ (2SD = 6.78). Comparing with the WHO 2007 growth standards [13], 72% of the adolescents had a BMI in the normal range. Fourteen percent of participants were overweight (pre-obese) while 6% were obese. These prevalence rates were greater in boys than in girls (P < 0.001), but the corresponding figure was not significant according to the age.

Table 1 presents the gender-specific height and BMI z-score statistics summary for the study participants according to the age group.

**Table 1:** **BMI-for-age and height-for-age z-score for Iranian students aged 10–14 and 15–19 years according to WHO growth standards 2007**[13]

**Gender**	**Age (year)**	**N**	**Height-for-age (%)**				**BMI-for-age (%)**						
			**<−3SD**	**<−2SD**	**Mean**	**SD**	**<−3SD**	**<−2SD**	**>+1SD**	**>+2SD**	**>+3SD**	**Mean**	**SD**
Combined	10-14	2460	2	6.1	−0.42	1.19	2	8.2	22.8	7.6	0.8	−0.10	1.42
	15-19	2970	1	4.3	−0.41	1.03	1.4	6.4	17.8	4.7	0.2	−0.15	1.26
Males	10-14	1250	1.7	6.3	−0.41	1.18	2.2	8.3	22.4	9.1	1.3	−0.12	1.05
	15-19	1465	1	5	−0.36	1.05	2.2	8.7	17.5	5.9	0.3	−0.24	1.34
Females	10-14	1210	2.4	6	−0.42	1.19	1.8	8.1	23.2	6.3	0.4	−0.09	1.37
	15-19	1505	1	3.7	−0.45	1.01	0.6	4.1	18.1	3.5	0.2	−0.06	1.18

SD: standard deviation ; N: The number of participants in each gender and age group; BMI: Body mass index; WHO: World Health Organization.

**Figure 1:**
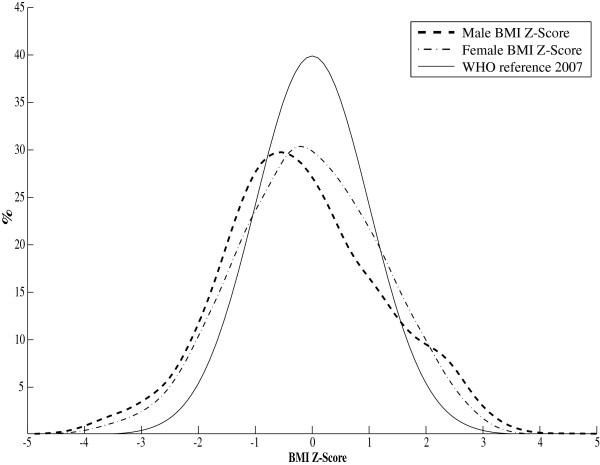
** BMI-for-age smoothed z-score histogram for Iranian children, aged 10–19 years.** (Male: dashed, Female: dash-dotted, and WHO reference 2007: solid line). The BCPE smoothing parameters were BCPE (λ=0.8, df(μ)=8, df(σ)=4, df(ν)=4, τ=2) for males and BCPE (λ=1, df(μ)=8, df(σ)=3, df(ν)=4, τ=2) for females

**Figure 2:**
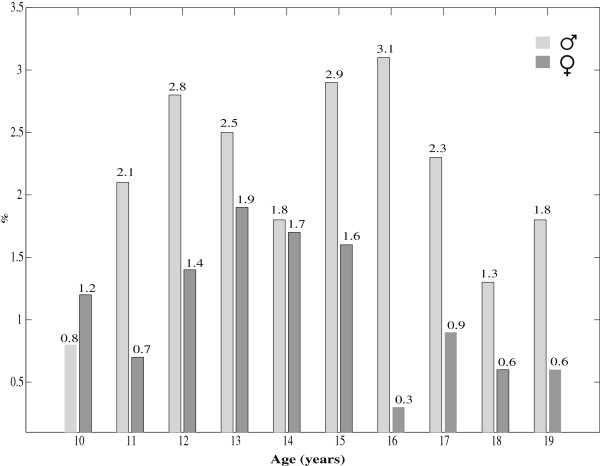
Prevalence of severe thinness (BMI z-scores < −3) among Iranian children, aged 10–19 years

**Figure 3:**
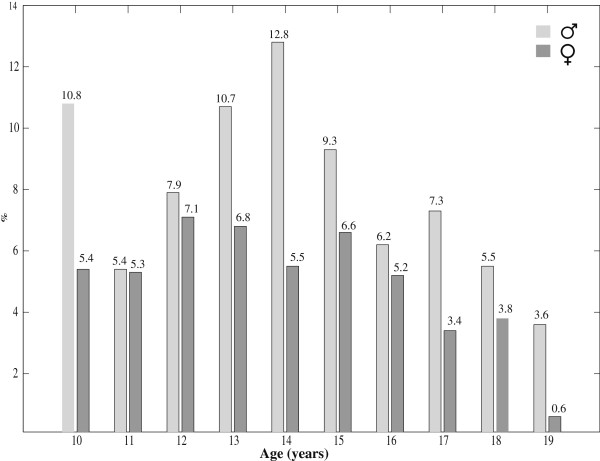
Prevalence of obesity (BMI z-scores>2) among Iranian children, aged 10–19 years

Figure 1 shows the BMI-for-age smoothed z-score histogram for Iranian adolescents of both genders in comparison with that of WHO 2007 reference curve [13]. Overall, 6% and 2% of participants were categorized as thin and severely thin, respectively. These frequencies were significantly different (P < 0.001) in terms of gender, with higher frequency of thinness and severe thinness among boys than girls. Table 2 presents the gender-specific average BMI-for-age of the Iranian children aged 10–19 years, is compared with that of WHO 2007 [13] and USCDC 2000 [14]. Significant differences (P < 0.001) were found for the BMI mean values when Iranian references were compared with the WHO 2007 [13] and USCDC2000 [14] growth charts. The mean BMI-for-age of the WHO 2007 and USCDC2000 references were greater than that of Iranian regional references. The prevalence of severe thinness (BMI z-scores < −3) and obesity (BMI z-scores >2) at different ages for Iranian adolescents are respectively depicted in Figures 2 and 3.

**Table 2:** **Body mass index of Iranian children, aged 10–19 years (CASPIAN-III Study) relative to the USCDC2000**[14]**and WHO2007**[13]**references**

**Reference**	**N**	**Mean**	**SD**	**Min**	**Max**
**Boys**					
**WHO 2007**	6227	19.33	2.04	16.40	22.20
**USCDC2000**	6227	19.50	2.01	16.62	22.48
**CASPIAN-III**	2715	19.02	1.26	17.75	21.31
**Girls**					
**WHO 2007**	6227	19.48	1.74	16.60	21.40
**USCDC2000**	6227	19.44	1.25	17.94	21.33
**CASPIAN-III**	2715	19.51	1.50	16.60	21.54

**Figure 4:**
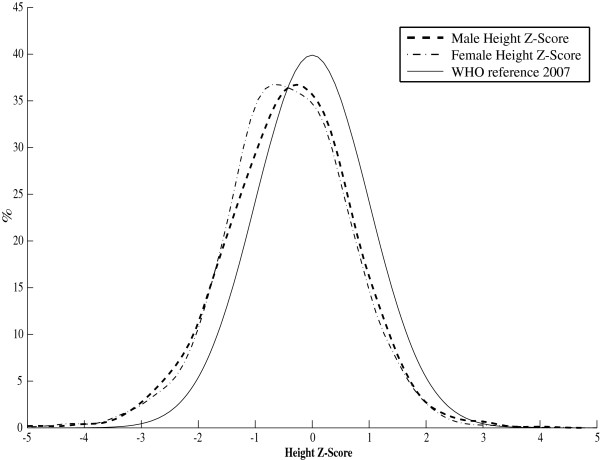
** Height-for-age smoothed z-score histogram for Iranian children, aged 10–19 years.** (Male: dashed, Female: dash-dotted, and WHO reference 2007: solid line). The BCPE smoothing parameters were BCPE (λ=1, df(μ)=12, df(σ)=4, df(ν)=1, τ=2) for males and BCPE (λ=0.85, df(μ)=10, df(σ)=4, df(ν)=1, τ=2) for females

**Figure 5:**
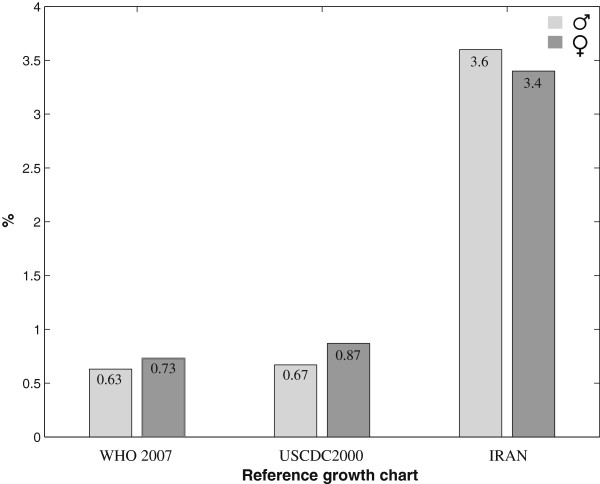
Prevalence of stunting (height z-scores < −2) among Iranian children, aged 10–19 years according to the WHO and USCDC references

The average height-for-age for Iranian adolescents was lower than the standard WHO and USCDC2000 mean references (Table 3).Stunting was found in 7% of Iranian adolescents, with higher prevalence among underweight males (52%) than their female counterparts (48%). The gender-specific smoothed z-score histogram of height-for-age among the studied individuals is depicted in Figure 4 in comparison with that of WHO2007 height reference curve [25]. The gender-specific prevalence of stunting in comparison with that of WHO2007 and USCDC2000 is shown in Figure 5. The percentage of stunted children aged 10–19 years, was higher than that of WHO 2007 [13] and USCDC2000 [14] in both genders.

**Table 3:** **Height of Iranian children, aged 10–19 years relative to the USCDC2000**[14]**and WHO2007**[13]**references**

**Reference**	**N**	**Mean**	**SD**	**Min**	**Max**
**Boys**					
**WHO 2007**	6227	161.89	14.52	137.80	176.50
**USCDC2000**	6227	162.26	14.45	138.61	176.60
**CASPIAN-III**	2715	155.80	12.89	144.23	174.02
**Girls**					
**WHO 2007**	6227	156.43	8.71	138.60	163.20
**USCDC2000**	6227	156.44	9.06	137.98	163.25
**CASPIAN-III**	2715	152.70	5.88	144.05	160.06

## Discussion

To the best of our knowledge, this study is the first of its kind in generating the WHO-based growth charts [13] for a nationally representative sample of children from the MENA region.

Comparison of the consistency of the growth charts of Iranian children aged 10–19 years with the international growth references revealed that the density of the tale distribution of boys/ girls BMI Z-scores are higher than that of WHO2007 (Figure 1), resulting in the existence of under- and over-weight children. This is in agreement with the findings that 8% and 21% of participants had low and high BMI respectively. Moreover, the boy/girl height smoothed Z-scores were shifted toward the left (Figure 4) indicating that there exist children whose height is below the normal range. This supports the findings that 7% were stunted.

Urbanization and nutritional transition as two important factors of epidemiological transition in Iran, similar to many other developing countries [31], are responsible for the emerging growth disorders in terms of underweight, overweight and obesity. Consumption of fat rich in saturated and trans-fatty acids has become a very popular component of everyday life in Iran which has undergone a rapidly occurring nutritional transition. Over-nutrition (overweight and obesity) and under-nutrition (thinness, sever thinness and stunting) do exist simultaneously in different age groups and also genders (Table 1; Figures 2 and 3).This could support that Iranian children aged 10–19 years are facing a double burden of growth disorders. This finding is consistent with our previous national report from Iran, as an upper-middle-income country [32].

Overweight and obesity are serious public health problems in many countries. Overweight affects 30%–80% of adults in the European countries. About 20% of children and adolescents are overweight, and a third of these are obese [33]. BMI is a widely used body weight classification system but has known restrictions, and may need to be adjusted for sitting height in order to be useful as an indicator of health risks in special populations. The BMI may overestimate the number of individuals that are overweight and obese, and hence at risk for type 2 diabetes mellitus and cardiovascular disease among the population [33]. In our study, the prevalence of overweight was 14.5% (13% for boys and 16% for girls, respectively) and the corresponding figure for obesity was 6% (8% for boys and 4% for girls, respectively). This result is considerably higher than the prevalence rate of 4% reported for China [34], but lower than 19.3% and over 20% found in Jamaica [35] and the United States of America [36], respectively. A study conducted in India [37] showed that the prevalence of obesity in affluent adolescent schoolchildren was 7.4%, and higher in males than in females similar to that of other study in Germany [38].

The maximum prevalence of obesity was found during the pubertal period ,i.e. between 10 to 12 years. This study showed that obesity was higher in boys than in girls. However, there are some documents that propose a higher prevalence of overweight and obesity in Iranian girls [39]. Being overweight adolescents, particularly boys, are more likely to have high serum cholesterol and abnormal lipoproteins levels in adulthood [40]. These consequences suggest an imbalance in the food intake of the population containing high energy foods especially carbohydrates and fats [41].

In the current study, the height of Iranian boys aged 14 years and above fell below references ranges. However, as much as (7%), 52% of the boys and 48% of the girls had height-for-age z-score values lower than -2SD of the WHO 2007 reference data. This disproportion in the prevalence of stunting in genders is in agreement with some other studies [42,43]. Some countries have high prevalence of stunting. For example rural Bangladesh has the highest prevalence of thinness and stunting (67% and 48% respectively) among adolescents. The 7% prevalence rate for adolescent was stunting as under- nutrition reported in this study is lower than the 18% reported for China [34] or 42.2% , 36.00% and 53.00% reported for adolescents in a Pakistani public school, Nepal and India respectively [37,43]. Twenty-five percent of the individual’s achieved height is attained during adolescence, which usually marks the end of growth [44]. The inferences of nutritional disadvantage for boys are unclear. Some studies showed that this issue is related to the boys’ delayed and longer growth spurt [4]. Stunting problem among girls has some important consequences in adulthood. A short woman tends to have a small pelvis and, therefore, is more likely to have hard delivery during childbirth [45].

The same trend was noticed for thinness and severe thinness as more boys than girls had z-score BMI-for-age values below -2SD and -3SD, respectively. Thinness may also limit school achievement and work productivity. Adolescents also provide a good proportion of the productive work force in such environments. Therefore, there is an urgent need to develop strategies to improve the growth and nutritional status of adolescents.

The main limitation of this study is that the national survey was cross-sectional. Future longitudinal studies considering the cardio-metabolic risk factors and genetic examinations should be accomplished to better characterize the nature of the nutritional transition documented in the current study. Moreover, we could not examine the pubertal status of the study participants.

In the meanwhile, the BMI cut-off values used to detect malnutrition was based on the universal indices proposed by WHO reference 2007. Some studies e.g. [46], showed that the malnutrition cut-offs are needed to be re-defined in some Asian countries [47]. The conclusion by the WHO [48] was that WHO BMI cut-off points should be retained as international classifications. However, there are some evidences that the ethnic differences must be taken into account. For example, global comparisons of the prevalence have showed Asians have ethnic predisposition to central body fat deposition and the metabolic syndrome even in a pediatric age [49,50]. Findings of our previous national study revealed ethnic differences in the cardio-metabolic risk factors ; it showed considerably higher prevalence of dyslipidemia, in terms of low HDL-C and hypertriglyceridemia in Iranian children and adolescents compared with their German and Brazilian counterparts [49,51]. These differences might be because of genetic-environment interactions. Future longitudinal studies considering biologic and biochemical risk factors can determine the impact of such ethnic differences in cardio-metabolic risk factors and the existing differences on the growth pattern of children in various populations on the incidence of CNCDs.

## Conclusions

The growth curves generated in this national study may be considered for establishing the new WHO global database on children’s growth. Based on the WHO reference 2007, over- and under-nutrition were found simultaneously in different age and gender categories. This nutritional transition must be considered in public health policy-making. The individual and public health effects of the differences in the growth charts of children in various populations should be determined in future longitudinal studies.

## Endnote

^a^Caspian is the name of the world's largest lake, located in Northern , Iran.

## Abbreviations

WHO: World Health Organization; BCPE: Box-Cox power exponential; CNCDs: Chronic non-communicable diseases; MGRS: Multi-centre Growth Reference Study; BMI: Body mass index; CASPIAN: **C**hildhood &**A**dolescence **S**urveillance and **P**revention of **I**ranian **A**dult **N**on communicable disease; MATLAB: Matrix Laboratory; US: United states; CDC: Centers for Disease Control and Prevention; SD: Standard deviation; NCHS: National Center for Health Statistics; BP: Blood pressure; HDL: High-density lipoprotein; HDL-C: The amount of cholesterol contained in High-density lipoprotein particles.

## Competing interests

The authors declare that they have no competing interests.

## Authors’ contributions

MM contributed to the study design, conducting the statistical analysis, and preparing the manuscript. HRM contributed to the study design, data analysis and editing the manuscript. RK contributed to design and conducting the study, interpretation of findings, and preparing the manuscript. MEM, TA, MT, RH, GA, and PP, contributed to the study design, conducting the study, and approving the manuscript. RM contributed to the study design, conducting the study, confirming the statistical analysis, and editing the manuscript. All authors read and approved the final version of the manuscript. Kelishadi R is the guarantor.

## Pre-publication history

The pre-publication history for this paper can be accessed here:

http://www.biomedcentral.com/1471-2431/12/149/prepub
